# Identification and Analysis of Alzheimer’s Candidate Genes by an Amplitude Deviation Algorithm

**DOI:** 10.4172/2161-0460.1000460

**Published:** 2019-02-02

**Authors:** Chaoyang Pang, Hualan Yang, Benqiong Hu, Shipeng Wang, Meixia Chen, David S Cohen, Hannah S Chen, Juliet T Jarrell, Kristy A Carpenter, Eric R Rosin, Xudong Huang

**Affiliations:** 1 College of Computer Science, Sichuan Normal University, Chengdu, China; 2 College of Mathematics and Software Science, Sichuan Normal University, Chengdu, China; 3 College of Management Science, Chengdu University of Technology, Chengdu, China; 4 Neurochemistry Laboratory, Department of Psychiatry, Massachusetts General Hospital and Harvard Medical School, Charlestown, MA, USA

**Keywords:** Alzheimer’s disease, Amplitude deviation algorithm, DNA microarray

## Abstract

**Background:**

Alzheimer’s disease (AD) is the most common form of senile dementia. However, its pathological mechanisms are not fully understood. In order to comprehend AD pathological mechanisms, researchers employed AD-related DNA microarray data and diverse computational algorithms. More efficient computational algorithms are needed to process DNA microarray data for identifying AD-related candidate genes.

**Methods:**

In this paper, we propose a specific algorithm that is based on the following observation: When an acrobat walks along a steel-wire, his/her body must have some swing; if the swing can be controlled, then the acrobat can maintain the body balance. Otherwise, the acrobat will fall. Based on this simple idea, we have designed a simple, yet practical, algorithm termed as the Amplitude Deviation Algorithm (ADA). Deviation, overall deviation, deviation amplitude, and 3δ are introduced to characterize ADA.

**Results:**

52 candidate genes for AD have been identified via ADA. The implications for some of the AD candidate genes in AD pathogenesis have been discussed.

**Conclusions:**

Through the analysis of these AD candidate genes, we believe that AD pathogenesis may be related to the abnormality of signal transduction (AGTR1 and PTAFR), the decrease in protein transport capacity (COL5A2 (221729_at), COL5A2 (221730_at), COL4A1), the impairment of axon repair (CNR1), and the intracellular calcium dyshomeostasis (CACNB2, CACNA1E). However, their potential implication for AD pathology should be further validated by wet lab experiments as they were only identified by computation using ADA.

## Background

### Introduction of Alzheimer’s disease and AD genes

Alzheimer’s disease (AD), the most common form of senile dementia, is a progressive neurodegenerative disorder characterized by global cognitive decline involving memory, orientation, judgment, and reasoning [1]. The disease itself was named after Alois Alzheimer [2], a Bavarian psychiatrist with expertise in neuropathology. According to statistics, AD is approaching epidemic proportions, with no cure or preventative therapy available [1]. By the year 2050, it is predicted that AD will affect 115.4 million people globally [3].

The AD pathogenesis is not yet well understood. In last 30 years, there have been many hypotheses trying to explain the cause of AD [1,2,4]. It is of common opinion that AD is related to amyloid plaques (Aβ) and neurofibrillary tangles (NFT) in the brain [1,2]. This hypothesis is supported by the location of the AD causative gene called the amyloid precursor protein (APP) on chromosome 21 [5,6]. In 2009, this theory was updated, suggesting that a close relative of the beta-amyloid protein, and not necessarily the beta-amyloid itself, may be the major culprit of the disease [7].

Three main genes associated with early onset AD are APP, PSEN1, and PSEN2 [8]. One main gene associated with late onset AD is APOE [8]. In 2009, evidence that attempted to prove ADAM10 as a candidate AD susceptibility gene was first provided [9]. Between 2009 and 2010, the genes PICALM, CL-U, CR1, and BIN1 were also put forward as potential AD candidate genes [10,11]. Other AD-related genes of interest such as CD33 and ATXN1 have also been identified [12–14].

### Introduction of DNA microarray and its application on AD genes

With little known about the cause of AD, it is necessary to identify more AD-related candidate genes. Prior to the year 2000, identifying AD candidate genes involved a large amount of time and money and yielded limited results. However, with the development of DNA microarray technology, researchers began to apply this technology in order to better identify potential AD genes [15].

DNA microarrays (also called gene chips) are a complex technology in molecular biology. A DNA microarray is typically a glass slide onto which DNA molecules are fixed in an orderly manner at specific locations called spots (or features). The DNA in a spot may either be genomic DNA or short stretches of oligonucleotide strands that correspond to a specific gene. The spots are printed onto the glass slide by a robot or are synthesized by the process of photolithography [16].

Researchers use DNA microarrays to measure the expression levels of thousands of genes simultaneously. Since DNA microarrays contain many spots, we can obtain many gene expression levels from a single experiment, compared to only being able to measure expression of one gene with a Northern Blot [17].

In order to analyze the DNA microarray data and identify AD candidate genes, researchers began designing computation methods (algorithms) in order to process the data. Currently, the most commonly used algorithms are the K-means Clustering algorithm [18], the Principal Component Analysis (PCA) algorithm [19], and the Ant Colony algorithm (ACO) [20].

### Organization of gene expression levels

In this manuscript, the original DNA microarray data were downloaded from the GEO Dataset within NCBI [21], which includes 22,283 genes. The data were obtained from control, incipient, moderate, and severe AD patients. All of these data are organized in a matrix format (Table 1). Table 1 consists of 22,283 rows and 9 columns, where the 22,283 rows correspond to the expression levels of the 22,283 genes, and the 9 columns represent the 9 samples (experiments). The matrix element in Table 1 comes from male controls, denoted by in this paper. The other three matrices, incipient, moderate, and severe, are denoted by, and, with 7, 8, and 7 columns, respectively.

## Methods

### The simple idea behind the amplitude deviation algorithm (ADA)

In order to identify AD candidate genes, we are proposing a new model based around the following principal: when an acrobat walks along a steel-wire, his/her body must have some swing; if the swing can be controlled by the individual then the acrobat can keep the body balance; otherwise, the acrobat will fall. Correspondingly, each gene can be seen as an acrobat and the change of expression level of each gene can be compared to the swing of an acrobat’s body on the tightrope. In the controlled stage (i.e., acrobats maintaining balance on the tightrope), since all genes have the ability of self-regulation, the gene expression levels are maintained within a certain range. However, when AD pathology develops in the brain, the expression levels of certain genes goes beyond the controlled range, analogous to the acrobats losing their balance. These are the genes that may be associated with AD.

### Data pre-processing

The data in the four different stages are organized as the matrices $T_{ctrol}\left(22,283^{⋆}9\right),T_{ircip}\left(22,283^{⋆}7\right),T_{moder}\left(22,283^{⋆}8\right),\text{and}\;T_{severe}\left(22,283^{⋆}7\right),$ respectively. Since the data of every column in each matrix is from one sample, the data in different columns are incomparable. In order to solve this problem, four data matrices are processed. In this paper, we process these matrices using the fact that the data in every column is equal to the value in the corresponding column minus that column’s average value. This column of differences then defines the difference as deviation:

(1) $$S\left(i,j\right)=T\left(i,j\right)-\frac{1}{m}\sum_{i=1}^{m}T\left(i,j\right),i=1,\ldots 22,283,j=1\ldots n,m=22,283,n=9;7;8;7$$

Where *s(I,j)* is defined as deviation matrix.

In the process of obtaining data, different experimental conditions (such as samples, equipment, temperatures, etc.) may generate errors. In order to reduce the potential errors, we calculated the average deviations for the different samples of the same stage, and this is how the overall deviation is calculated. The format of is presented as follows:

(2) $$D\left(i\right)=\frac{1}{n}\sum_{j}^{n}S\left(i,j\right)$$

i=1,2,…,22283;

n=9,7,8,7.

### Mathematical representation of the ADA

In this paper, each gene is compared to an acrobat as aforementioned. When an acrobat walks along a tightrope, his body must have some swing. Correspondingly, there must be certain changes of expression levels of each gene. More specifically, for each gene there must be differences between the controlled stage and the incipient, moderate, and severe stages. We use these differences to characterize the changes. Then the deviation amplitude is introduced (here, deviation amplitude can be interpreted as the difference). The format of deviation amplitude is presented as follow:

(3) $$A_{incip}\left(i\right)=D_{incip}\left(i\right)-D_{ctrol}\left(i\right)$$

(4) $$A_{moder}\left(i\right)=D_{moder}\left(i\right)-D_{ctrol}\left(i\right)$$

(5) $$A_{severe}\left(i\right)=D_{severe}\left(i\right)-D_{ctrol}\left(i\right)$$

Where *D_cotrl_*(*i*),*D*_*invip*_(*i*),*D*_*moeder*_(*i*),*D*_*severe*_(*i*), are denoted by the overall deviation in four stages and *A*_*incip*_(*i*),*A*_*moder*_(*i*),*A*_*severe*_(*i*) represents the deviation amplitude in the incipient, moderate and severe stage, respectively.

Here, we find that *A*_*ircip*_(*i*) satisfies the normal distribution via the corresponding statistical histogram, shown in Figure 1A. Similarly, both *A*_*moder*_(*i*) and *A*_*severe*_(*i*) satisfy normal distribution (their corresponding averages are almost equal to 0, their variances are equal to 0.417 and 0.536, respectively).

The normal distribution follows the 3σ principle, which asserts that 99.7% of the data falls within a range of 3σ. Any sample that does not follow this principle is abnormal. We use the 3σ principle as the criterion for characterizing how big of a range our “acrobats” can have while maintaining stability. That is to say, when the change of expression level of a gene is greater than 3σ or less than −3σ (i.e., $A_{t}\left(t\right)-{\bar{A}}_{t}|>3\sigma$, *t* represents the different stages) and its overall deviation is consistently and significantly upregulated or downregulated, the gene is a candidate for AD.

The computation was performed, and 12 genes were identified as AD candidate genes (Table 2). Among them are 7 genes whose average deviations are significantly and consistently upregulated (listed in the right column of Table 2), and 5 genes whose average deviations are significantly and consistently downregulated (listed in the left column of Table 2).

This result is not ideal. In addition to the small number of AD candidate genes, the data collected was affected by noise during the experimental process. If we can keep the original basis of the result and relax the conditions appropriately, the effects should improve. Therefore, the specific ADA is formulated:

Below, deviation amplitude is defined as the forms of eqns. (6), (7) and (8):

(6) $$A_{incip}\left(i\right)=\left|D_{incip}\left(i\right)-D_{ctrol}\left(i\right)\right|i=1,\ldots 22283$$

(7) $$A_{moder}\left(i\right)=\left|D_{moder}\left(i\right)-D_{ctrol}\left(i\right)\right|i=1,\ldots 22283$$

(8) $$A_{severe}\left(i\right)=\left|D_{severe}\left(i\right)-D_{ctrol}\left(i\right)\right|i=1,\ldots 22283$$

The statistical histogram of *A*_*incip*_(*i*) is shown in Figure 1B. Here, *A_incip_*(*i*) does not obey the normal distribution. Both *A*_*moder*_(*i*) and *A*_*severe*_(*i*) also do not satisfy normal distribution.

In statistics, σ has a common formula as follows:

$$\sigma =D\left(x\right)=E{\left(x-x\right)}^{2}=E\left(x^{2}\right)=E\left(x^{2}\right)-{\left[E\left(x\right)\right]}^{2}$$

Where *E*(*x*), *D*(*x*) is the expectation and variance of *x* respectively.

When the deviation amplitude is defined as eqn. (3), the value of *E*(*x*) is almost equal to zero. However, when the deviation amplitude is defined as eqn. (6), the value of *E*(*x*) is greater than zero, which leads to a decreased value of *D*(*x*). Correspondingly, 3σ gets smaller as well. *via* $A_{t}\left(t\right)-{\bar{A}}_{t}\left|>3\sigma >3\sigma^{*}\left(\sigma^{*}\right)\right.$ represents the variance which are from eqns (6), (7) and (8)), 3*σ*^⋆^ is selected to be the biggest threshold of range that allows acrobats to keep their balance. Since *A*_*t*_(*i*)(t represents the different stages) does not obey the normal distribution, we propose the following two criterions to identify AD candidate genes:

Criterion 1:

(9) $$A_{incip}\left(i\right)>3{\sigma^{⋆}}_{incip}>3{\sigma^{⋆}}_{moder}>3{\sigma^{⋆}}_{severe}$$

(10) $$D_{ctrl}\left(i\right)<D_{incip}\left(i\right)<D_{moder}\left(i\right)<D_{severe}\left(i\right)$$

Criterion2:

(11) $$A_{incip}\left(i\right)>3\sigma_{incip}^{{}^{*}},A_{moder}>3\sigma_{moder}^{{}^{*}},A_{severe}$$

(12) $$D_{ctrl}\left(i\right)>D_{incip}\left(i\right)>D_{moder}\left(i\right)>D_{severe}\left(i\right)$$

Implementation of the ADA to identify AD candidate genes

Step 1: Use eqn. (1) to calculate deviation matrices in four different stages

Step 2: Use eqn. (2) to calculate the overall deviation of each gene in four stages

Step 3: Use eqns. (3), (4) and (5) to calculate three deviation amplitudes of each gene (incipient, moderate and severe stages).

Step 4: Use eqns. (6), (7) and (8) to calculate three deviation amplitudes of each gene (incipient, moderate and severe stages).

Step 5: Use eqns. (9), (10), (11) and (12) to identify AD candidate genes.

## Results

Here, we identify 52 candidate genes. Out of these genes, 27 have deviation amplitudes with consistent rises (Figure 2A), and 25 genes have deviation amplitudes with consistent declines (Figure 2B). It is worth noting that the 52 genes discovered with the ADA also contain formerly identified AD candidate genes. Thus, the results of the second set of equations not only retain the original results, but also achieve the conditions of relaxation; therefore, the second set should be better at reflecting the actual number of AD-related gene expressions. Tables 3 and 4 contain candidate genes selected by the first and second criterion.

## Discussion

### Analysis of AD candidate genes

The healthy human body maintains physiological homeostasis in countless aspects, and this balance depends on coordination among proteins in the human body. Proteins are subject to the regulation of gene expression, so the coordination among proteins depends on the coordination of gene expression levels. Once the coordination of gene expression levels is destroyed, the body will enter pathophysiological status, resulting in disease. Since AD is a chronic neurodegenerative disorder that is characterized by memory impairment, cognitive dysfunction, and behavioral disturbances, this paper claims that AD revolves around a nervous system imbalance, which is associated with an imbalance of gene expression levels. Therefore, these genes whose expression levels are out of balance are AD candidate genes.

The ADA proposed in this paper attempts to find those genes with dramatic changes in gene expression levels, which then leads to an imbalance. It is possible that this imbalance may either be the cause or outcome of AD.

After analyzing the locations and functions of the proteins encoded by the identified genes, the following features were discovered and certain new pathological factors of AD were conjectured. First, most of the proteins encoded by these identified genes are located in the membrane and the cytoplasm (the distributions of proteins are shown in Figure 3A).

The functions of most proteins encoded by these identified genes correlate with signal transduction, metabolism, regulation of transcription, protein transport, immune response, and protein degradation (especially regarding signal transduction, metabolism, regulation of transcription, and transport (Figure 3B). The location and functions of these proteins suggests that analyzing the proteins at the membrane and the cytoplasm is helpful for exploring the AD pathology.

Moreover, proteins located specifically on membranes are mainly involved in signal transduction and protein transport (Figure 3C), and the proteins located specifically at the cytoplasm mainly correlate with protein transport and degradation (Figure 3D). Based on these findings, we can infer that the factors causing AD are significantly associated with signal transduction, metabolism, regulation of transcription, and protein transport and degradation. Loss of proper signal transduction and signaling pathway function [22,23], loss of regulation of transcription, [24] and dysregulation of metabolism [25] have all been correlated with AD progression in previous studies.

**Figure 4:**
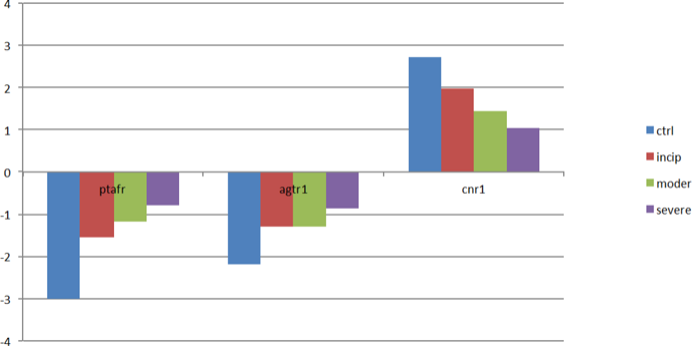
The expression levels of genes (AGTR1, PTAFR, CNR1) with the deterioration of AD. Genes AGTR1 and PTAFR are associated with signal transduction. With the AD progression, their expression levels are significantly upregulated. Inversely, the expression level of gene CNR1 is downregulated.

In regard to particular genes studied, the abnormal expression levels of genes (AGTR1 and PTAFR) are associated with increasing protein kinase C (PKC), which can promote the accumulation of amyloid Aβ, potentially leading to AD. The protein AGTR1 (angiotensin II receptor 1), encoded by gene AGTR1, allows for binding of angiotensin II, which generates diacylglycerol, and in turn activates PKC [26] which markedly decreases Aβ release from cells [27]. Since the expression level of gene AGTR1 consistently increases with AD progression (Figure 4), the excessive cellular accumulation of Aβ will be promoted, which may lead to AD [7]. There is evidence to suggest that angiotensin II receptor blockers may be a viable option for AD treatment [28], and this may be because these blockers prevent PKC from decreasing amyloid Aβ release.

The increase of the protein PTAFR (platelet-activating factor, PAF), a phospholipid signaling molecule, causes increased binding to its corresponding receptor (RAF-R) in the membrane surface which can activate phosphatidylinositol and phospholipase C [7]. In the phosphatidylinositol pathway, extracellular signaling molecules which bind G protein-coupled receptors on the cell surfaces cause the hydrolysis of phosphatidylinositol diphosphate into two products: inositol triphosphate (IP3e) and diacylglycerol (DG). DG can activate protein kinase c [29]. Protein kinase c markedly decreased the Aβ release from cells, [27] and increases the accumulation of Aβ that may lead to AD [7]. A recent study suggests that aberrant lipid signaling is correlated with AD [30].

Furthermore, the abnormal expression of gene CNR1 correlates with the absence of LTD (long-term depression), which may lead to the impairment of LTP (long-term potentiation) and then may induce AD. The protein CNR1 (Cannabinoid receptor 1), which is mainly distributed in the central nervous system (CNS), is involved in preventing neurotransmitter release. Since the protein CNR1 is significantly downregulated in the presence of AD, (Figure 4), LTD is not activated [31,32]. The absence of LTD may contribute to abnormalities of learning and memory-related behavior [33], which are AD symptoms.

Additionally, the abnormal expressions of genes (COL5A2 (221729_at), COL5A2 (221730_at), COL4A1) are associated with a decrease in energy supply, which then can lead to neuronal apoptosis, an AD neuropathological feature [34]. All of the proteins encoded by genes COL5A2 (221729_at), COL5A2 (221730_at), and COL4A1 are involved in phosphate transport in an organism’s activities. Phosphorus is one of the main elements that make up the human body; it is largely involved in the body’s energy metabolism and it is also an important component of adenosine triphosphate (ATP) [35]. A recent study revealed that higher levels of serum phosphorus are correlated with increased risk for dementia [36]. More research is needed to find out more information about the connection between phosphorus and AD.

**Figure 5:**
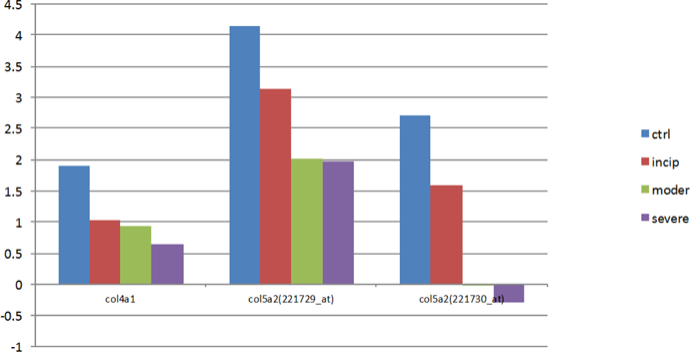
The expression levels of genes (COL5A2 (221729_at), COL5A2 (221730_at), COL4A1). Genes (COL5A2 (221729_at), COL5A2 (221730_at), COL4A1) are related to transport. With the AD progression, their expression levels are all downregulated.

Figure 5 explains that the expression of proteins encoded by these genes (COL5A2 (221729_at), COL5A2 (221730_at), COL4A1) is consistently downregulated, which may contribute to energy metabolism disorders and then affect a series of activities such as signal transduction, transcription, protein degradation and transport, which, as mentioned previously, are related to AD. Neuronal apoptosis, another AD pathological feature mentioned previously, may be the result of this downregulation.

### Analysis of candidate genes associated with metal ions

In the 52 identified genes, 14 genes (about one-third of the total) are associated with metal ions. After analyzing these metal ion genes, the following characteristics are observed. First, most of the genes associated with metal ions are related to calcium ions (Figure 6A). As shown in Figure 6A, 14 genes are mainly related to calcium ions. Numerous studies have shown there is a close relationship between calcium dyshomeostasis and AD [37–39]; therefore, it is necessary to study these genes associated with calcium ions. In fact, a recent study that added new evidence for the Calcium Hypothesis of Alzheimer’s and Brain Aging details how changes in calcium signaling can affect neurons and, in some cases, promote death and disease [40]. Clearly, the connection between AD and calcium has been an active area of research for decades; since calcium plays such a significant role in neuronal function, it is no surprise that dysregulation of calcium may promote AD.

After analyzing the genes associated with calcium ions, we saw that most of the proteins encoded by these genes are distributed in the membrane and extracellular regions. The percentage of protein distributions are shown in Figure 6B. In addition, we found that proteins encoded by the genes associated with calcium ions are mainly involved in signal transduction and metabolism. The percentages of protein functions are shown in Figure 6C.

**Figure 7:**
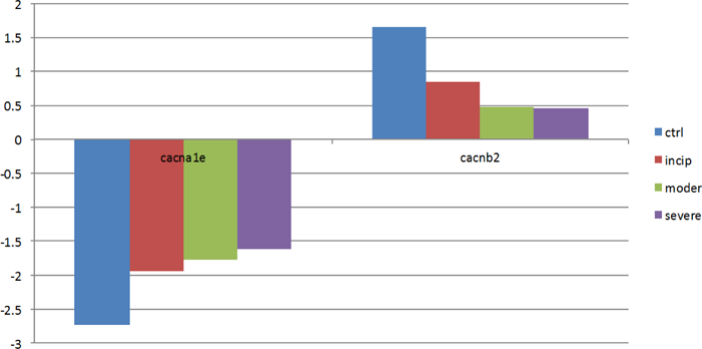
The expression levels of genes (CACNB2, CACNA1E). Genes (CACNB2, CACNA1E) are associated with calcium ions. As the severity of AD increases, the expression levels change sharply.

We also investigated the CACNB2 and CACNA1E proteins, which are the β2 subunit and α1E subunit, respectively, of a voltage-dependent calcium channel. The protein voltage-dependent calcium channel (voltage-dependent calcium channels, VDCC), which is located in the cell membrane, controls the intake of calcium ions into the cell. VDCC activity is determined by the α1 subunit whose functions are regulated by a β subunit (β1-β4) [41]. Since the expression level of gene (CACNB2) is significantly downregulated with the deterioration of AD (Figure 7), the ability of a β2 subunit to regulate an α1subunit may weaken, which may cause the expression of the gene CACNA1Ecacna1e to consistently increase with AD progression (Figure 7). The increasing proteins encoded by the gene cacn1e may induce VDCC activity changes. Moreover, VDCC activity changes may cause a change in calcium influx, which may lead to intracellular calcium dyshomeostasis and induce AD. This conclusion coincides with the idea that AD is correlative with the intracellular calcium dyshomeostasis [39].

### Potential implications for the AD candidate genes

**Figure 8:**
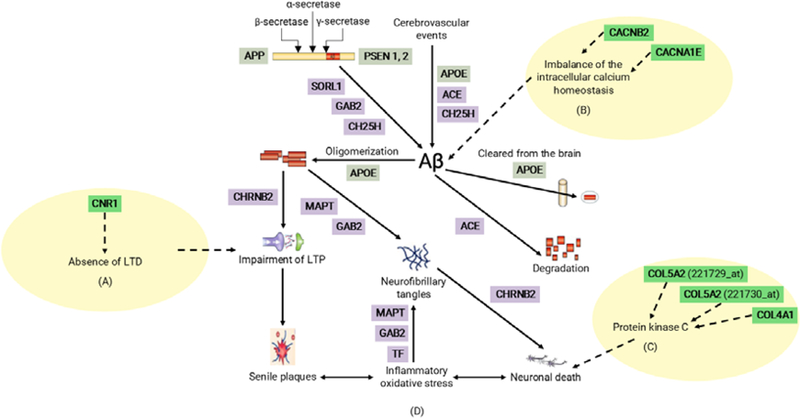
Summary of possible pathogenetic roles for AD candidate genes identified in this paper. Part A, B, and C are added to be four possible pathogenetic roles and are highlighted with a dashed line. Part D is from Figure 1 in reference [2].

In this paper, we studied eight genes selected from a list of identified AD candidate genes and discussed their potential implications in AD. Although we believe that these AD candidate genes are interesting for many reasons, including the dramatic change of gene expressions and its proven role in AD pathogenesis by other references (Figure 8), the potential implication should be used only as a reference.

## Conclusions

Even though AD is the most common form of dementia, its pathological mechanisms are not fully revealed. However, three early-onset familial AD genes (APP, PSEN1, and PSEN2) and one genetic risk factor for late-onset AD (APOE) have been identified. With the applications of DNA microarray technology, identifying more AD candidate genes by computation appears particularly promising.

By using ADA, we were able to identify 52 genes that showed dramatic changes in gene expression, and thus can be identified as potential AD candidate genes. With regards to these candidate genes, 27 genes showed average amplitudes with unanimous rises (Figure 2A) and 25 genes showed average amplitudes that consistently downregulated with the deterioration of AD (Figure 2B).

By studying these AD candidate genes, the following four pathogenetic roles are determined: (1) the abnormal expression levels of genes (AGTR1 and PTAFR) are associated with an increase in the activity of protein kinase c, which promotes the accumulation of Aβ, in turn leading to AD; (2) The abnormal expression of gene CNR1 correlates with the absence of LTD, which may lead to the impairment of LTP, and subsequently may induce AD; (3) The abnormal expressions of genes (COL5A2 (221729_at), COL5A2 (221730_at), COL4A1) are associated with decreases in energy supply, which may lead to neuronal apoptosis, a pathological feature of AD; (4) The abnormal expressions of genes (CACNB2, CACNA1E) correlate with the intracellular calcium dyshomeostasis, which is related to AD.

Based upon this study, we propose that AD pathogenesis may be related to abnormality of signal transduction (AGTR1 and PTAFR), decrease in protein transport capacity (COL5A2 (221729_at), COL5A2 (221730_at), COL4A1), impairment of axon repair (CNR1), and intracellular calcium dyshomeostasis (CACNB2, CACNA1E).

Finally, since these AD candidate genes were only identified by computation using ADA their potential implication for AD pathology should be further validated by wet lab experiments.

## Figures and Tables

**Figure 1A:**
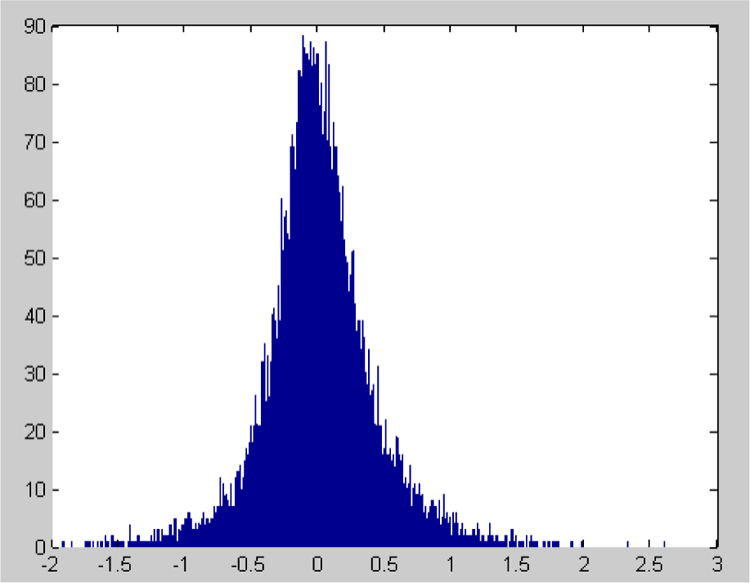
The statistical histogram of *A*_*incip*_(*i*) and shows that the deviation amplitude *A*_*incip*_(*i*) obeys the normal distribution (the average value of *A*_*incip*_(*i*) is approximately 0 and the variance is equal to 0.3609).

**Figure 1B:**
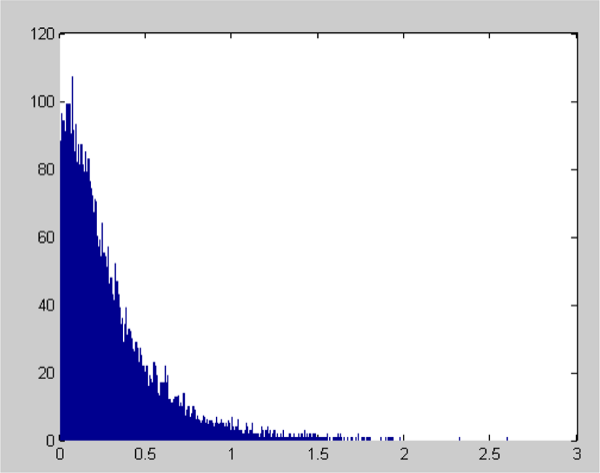
The statistical histogram of *A*_*incip*_(*i*) and indicates that *A*_*incip*_(*i*) doesn’t obey normal distribution.

**Figure 2A:**
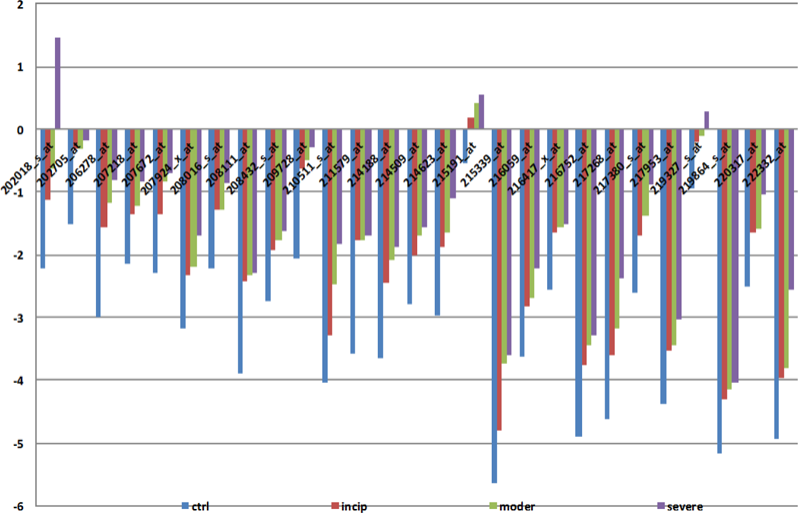
AD candidate genes with consistent rises in deviation amplitude. Figure 2A shows that 27 genes out of 52 AD candidate genes have deviation amplitudes with consistent rises.

**Figure 2B:**
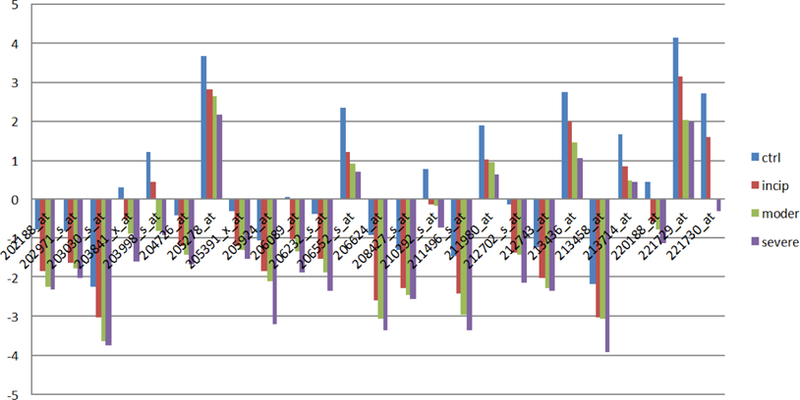
AD candidate genes with consistent declines in deviate amplitude and shows that 25 genes out of 52 AD candidate genes have deviation amplitudes with consistent declines.

**Figure 3A:**
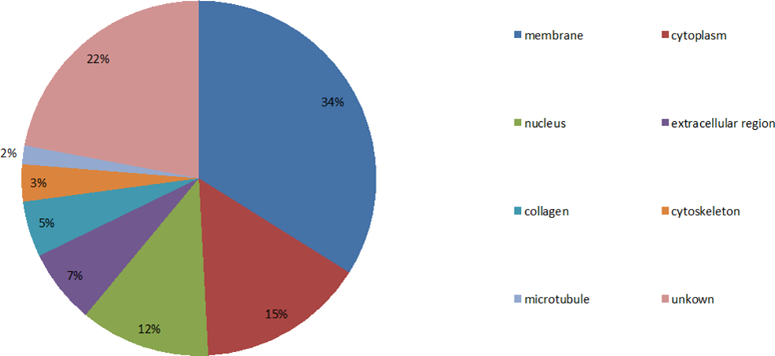
Distribution of proteins encoded by these identified genes and 34% of proteins are located in the membrane, 15% at the cytoplasm, 12% at the nucleus, 7% at an extracellular region, 5% at the collagen, 3% at the cytoskeleton, 2% at the microtubule, and 22% at other areas. The results suggest that potential causative factors of AD pathology may be associated with the proteins located at the membranes of neurons, cytoplasms, and nuclei and extracellular regions (particularly at the membrane and cytoplasm).

**Figure 3B:**
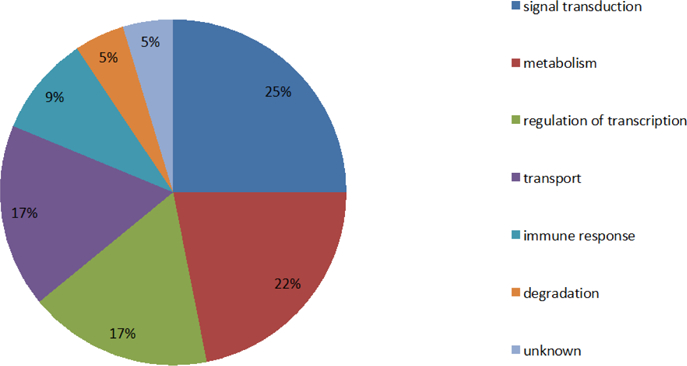
Distribution ratios of protein functions and shows that the percentages of proteins involved in signal transduction, metabolism, regulation of transcription, and protein transport, are 25%, 22%, 17%, and 17%, respectively, which are greater than the percentages of proteins associated with immune response and protein degradation.

**Figure 3C:**
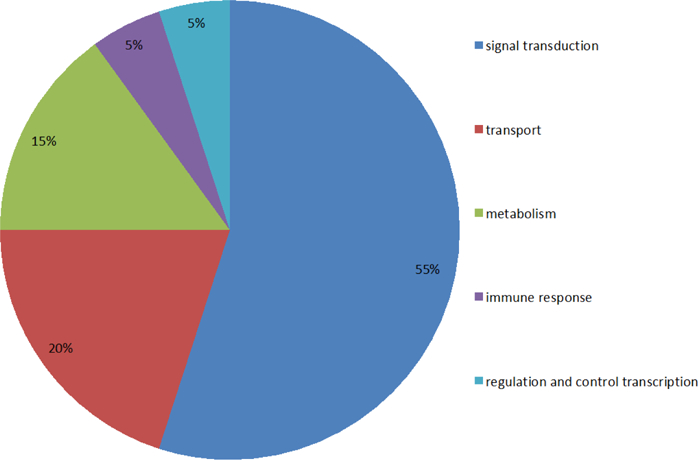
Percentages of functions of membrane proteins and shows that the membrane proteins associated with signal transduction, protein transport, metabolism, immune response, and regulation/ control of transcription are respectively 55%, 20%, 15%, 5% and 5%.

**Figure 3D:**
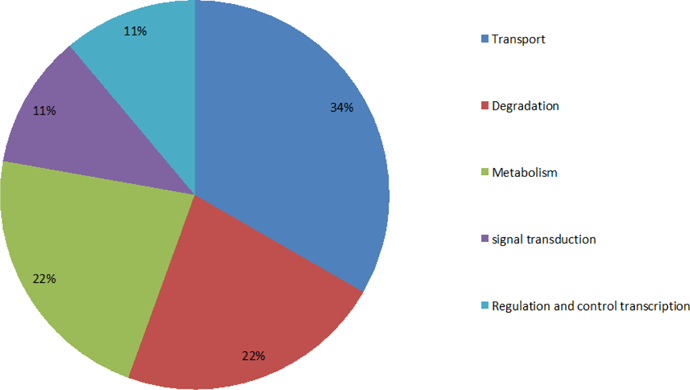
The functions of proteins in the cytoplasm and shows the percentages of proteins involved in protein transport and degradation, metabolism, signal transduction, and regulation/control of transcription are 34%, 22%, 22%, 11% and 11%, respectively.

**Figure 6A:**
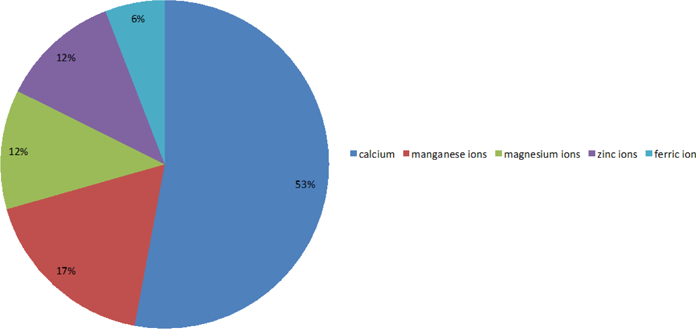
The proportions of genes associated with various metal ions and the percentages of genes associated with calcium ions, manganese ions, magnesium ions, zinc ions, and ferric ions are 53%, 17%, 12%, 12%, and 6%, respectively.

**Figure 6B:**
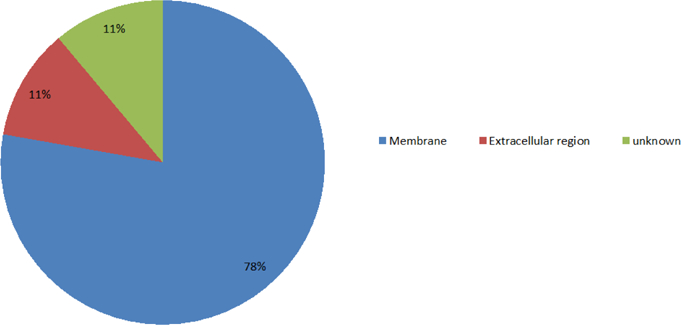
The distributions of proteins encoded by the calcium-related genes. The percentages of proteins located at the membrane, the extracellular region, and unknown are, 78%, 11%, and 11%, respectively.

**Figure 6C:**
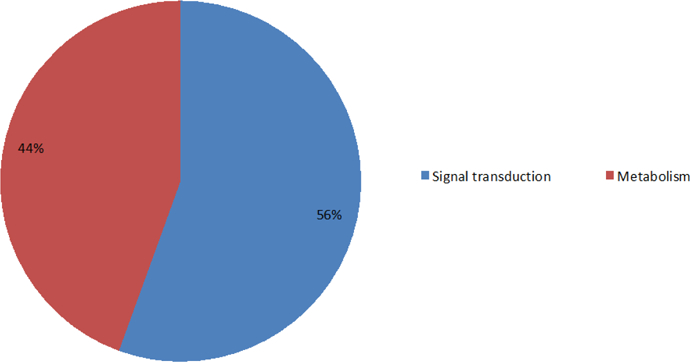
The percentages for functions of proteins encoded by the genes associated with calcium ions. 56% are involved in signal transduction, 44% are related to metabolism.

**Table 1:** The organization of gene expression levels.

		1st col.	2nd col.	…	9th col.
Gene No.	AFFX-NAME	GSM 21215	GSM 21217	…	GSM 21232
1	BioB-5_at	8.937	9.941	…	9.386
2	BioB-M_at	9.278	10.56	…	10.37
3	BioB-3_at	7.9	9.033	…	9.299
4	BioC-5_at	10.18	11.46	…	10.91
…	…	…	…	…	…
22283	222384_at	5.092	6.463	…	6.6

Note 1: In Table 1, each data (gene expression level) has been log-transformed but not normalized along the column.

**Table 2:** The candidate genes identified by 3σ principle.

Affymetrix Probe Set Name	Symbol	Affymetrix Probe Set Name	Symbol
202018_s_at	LTF	206232_s_at	B4GALT6
206278_at	PTAFR	206552_s_at	TAC1
209728_at	HLA-DRB4	206624_at	USP9Y
211579_at	ITGB3	212702_s_at	BICD2
214188_at	HEXIM1	221730_at	COL5A2
214623_at	FBXW4P1		
216752_at	PIK3R4		

**Table 3:** The AD candidate genes selected by the first criterion.

Gene ID	Abbreviation	Gene ID	Abbreviation	Gene ID	Abbreviation
202018_s_at	LTF	216059_at	PAX3	209728_at	HLA-DRB4
208016_s_at	AGTR1	216417_x_at	HOXB9	210511_s_at	INHBA
208432_s_at	CACNA1E	216752_at	PIK3R4	211579_at	ITGB3
215191_at	THOP1	217268_at	RAB7A	214188_at	HEXIM1
207218_at	F9	217380_s_at	XPNPEP1	214509_at	HIST1H3I
207672_at	RFX4	217953_at	PHF3	222332_at	HG-U133A
207924_x_at	PAX8	219327_s_at	GPRC5C	214623_at	FBXW4P1
208111_at	AVPR2	219864_s_at	RCAN3	206278_at	PTAFR
202705_at	CCNB2	220317_at	LRAT	215339_at	NKTR

**Table 4:** The AD candidate genes selected by the second criterion.

Gene ID	Abbreviation	Gene ID	Abbreviation	Gene ID	Abbreviation
202188_at	NUP93	208427_s_at	ELAVL2	206089_at	NELL1
202971_s_at	DYRK2	210292_s_at	PCDH11Y	206232_s_at	B4GALT6
203030_s_at	PTPRN2	211980_at	COL4A1	206552_s_at	TAC1
203841_x_at	MAPRE3	212702_s_at	BICD2	206624_at	USP9Y
203998_s_at	SYT1	212743_at	RCHY1	221729_at	COL5A2
204726_at	CDH13	213436_at	CNR1	221730_at	COL5A2
205278_at	GAD1	213458_at	FAM149B1	211496_s_at	PDC
205391_x_at	ANK1	213714_at	CACNB2		
205924_at	RAB3B	220188_at	JPH3		

## Abbreviations:

AD: Alzheimer’s Disease
ADA: Amplitude Deviation Algorithm
Αβ: Abeta Amyloid Peptide
NFT: Neurofibrillary Tangles
APP: Amyloid Beta Precursor Protein
PCA: Principal Component Analysis
ACO: Ant Colony Algorithm
PAF: Platelet-Activating Factor
PKC: Protein Kinase C
IP3e: Inositol Triphosphate
DG: Diacylglycerol
CNR1: Cannabinoid Receptor 1
LTD: Long-Term Depression
LTP: Long-Term Potentiation
ATP: Adenosine Triphosphate
VDCC: Voltage-Dependent Calcium Channels
